# The specialized competency framework for community pharmacists (SCF-CP) in Lebanon: validation and evaluation of the revised version

**DOI:** 10.1186/s40545-023-00585-6

**Published:** 2023-06-21

**Authors:** Fouad Sakr, Marwan Akel, Hala Sacre, Chadia Haddad, Samah Tawil, Jihan Safwan, Aline Hajj, Rony M. Zeenny, Katia Iskandar, Pascale Salameh

**Affiliations:** 1 School of Pharmacy, Lebanese International University, Beirut, Lebanon; 2 INSPECT-LB (Institut National de Santé Publique, d’Épidémiologie Clinique Et de Toxicologie-Liban), Beirut, Lebanon; 3 School of Education, Lebanese International University, Beirut, Lebanon; 4 Gilbert and Rose-Marie Chagoury School of Medicine, Lebanese American University, Byblos, Lebanon; 5 School of Health Sciences, Modern University of Business and Science, Beirut, Lebanon; 6 Research Department, Psychiatric Hospital of the Cross, Jal El Dib, Lebanon; 7 Laboratoire de Pharmacologie, Faculty of Pharmacy, Pharmacie Clinique et Contrôle de Qualité Des Médicament (LPCQM), Saint Joseph University of Beirut, Beirut, Lebanon; 8 Faculté de Pharmacie, Université Laval, Québec, Canada; 9 Oncology Division, CHU de Québec Université Laval Research Center, Québec, Canada; 10 Department of Pharmacy, American University of Beirut Medical Center, Beirut, Lebanon; 11 Faculty of Pharmacy, Lebanese University, Hadat, Lebanon; 12 University of Nicosia Medical School, Nicosia, Cyprus

**Keywords:** Community pharmacy, Framework, Lebanon, Pharmacist, Specialized competency

## Abstract

**Background:**

In the absence of similar studies in Lebanon, this study aimed at upgrading and validating the Lebanese specialized competencies framework for community pharmacists (SCF-CP) as a tool to transform community practice and support the professional development and career progression of community pharmacists.

**Methods:**

Content validity was assessed and improved through a team of experts. After a thorough literature review and utilizing the Delphi technique, six domains were defined in the framework, with their respective competencies and behaviors. A cross-sectional study was then carried out from March to October 2022 using an online questionnaire created on Google Forms. The snowball technique was applied to reach community pharmacists across all the Lebanese governorates.

**Results:**

The final sample included 512 community pharmacists. The construct validity of the framework was confirmed by factor analysis. The Kaiser–Meyer–Olkin measures of sampling adequacy were satisfactory for all models ranging from 0.500 to 0.956 with a significant Bartlett’s test of sphericity (*P* < 0.001). The internal consistency of all competency domains was confirmed by Cronbach’s alpha, with values ranging from 0.803 to 0.953. All competencies were significantly correlated with their respective domains (*P* < 0.001), and all domains were significantly correlated with each other and with the framework (*P* < 0.001). The participants declared being competent in all domains relating to fundamental skills, safe and rational use of medicines, pharmacy management, professional skills, public health fundamentals, and emergency preparedness and response, with some exceptions, such as compounding, management, and emergency preparedness. A higher declared competency level was associated with having more experience and receiving more than 50 patients per day.

**Conclusion:**

Our findings could demonstrate that the Lebanese specialized competency framework is a valid and reliable tool. This framework could help assess the minimum competencies that community pharmacists should possess or acquire and direct initial and continuing education for better practice. Hence, it could be adopted by the authorities and implemented in the Lebanese community pharmacy setting.

**Supplementary Information:**

The online version contains supplementary material available at 10.1186/s40545-023-00585-6.

## Background

The roles of healthcare professionals are rapidly changing worldwide, placing additional demands on the healthcare professions, including pharmacy. In many countries, the expanded patient-centered roles for pharmacists have been recognized through a wide range of national healthcare system reforms [1]. As new technologies are added to therapeutic care, pharmacists must acquire new knowledge, skills, and behaviors to be able to apply new modalities to patient care [2]. Furthermore, pharmacists are no longer solely responsible for drug dispensing; they now play an essential role in community health and wellness through expanded services, such as educational counseling [3], medication safety [4], and medication reconciliation and management [5, 6].

Community pharmacists are the most accessible healthcare professionals, with capabilities in different therapeutic areas such as drug information and health promotion. They are in a unique position to bring drug knowledge to the community [7]. With this responsibility, the competencies of community pharmacists should consistently be assessed according to a specific framework where the development of an adaptable and accessible healthcare workforce is imperative to build and maintain resilient health systems and improve the healthcare services delivered [8, 9]. In this perspective, regulatory bodies such as the International Pharmaceutical Federation (FIP) and the World Health Organization (WHO) designed a pharmacy education action plan that focused on identifying local needs and adapting educational programs [10–12]. This plan pertains to a subset of outcomes, including knowledge, skills, and attitudes, which relate to professional performance and focus on improvement through training and development [13].

At the global level, a profession-wide competency framework for foundation-level pharmacists was developed based on the FIP Global Competency Framework, but it was not specific to community pharmacists in particular [14, 15]. Many developed countries have established a competency framework intended for the practice of community pharmacists and recognized it as a means to facilitate their continuing professional development (CPD) and performance review [16]. However, developing countries do not yet have such a framework. In Malaysia, a survey exploring patient-oriented services provided by community pharmacists beyond processing prescriptions and dispensing medications revealed a trend toward the allocation of such activities, but this practice was not widely implemented [17]. Thus, given the fundamental role of pharmacy education and CPD in preparing and producing competent pharmacists in Arab countries, a national competency framework for pharmacists is necessary to bridge the gap between traditional pharmacy education and the challenging demands of modern health systems.

Following a similar approach, the Order of Pharmacists of Lebanon (OPL, the official national pharmacy association) suggested assessment projects related to developing a validated core competency framework for both pharmacy graduates and practicing pharmacists in Lebanon based on international frameworks [18, 19]. The OPL also suggested specialized frameworks for various pharmacy practice fields, including community pharmacy [20]. However, to date, this framework has not been validated nor implemented, and it remains unknown whether community pharmacists have the minimum competencies required to be held accountable in their practice field. Thus, it was deemed necessary to review and update the specialized competency framework that was designed specifically for community pharmacy practitioners.

Therefore, this study aimed to revise and validate the Lebanese specialized competency framework for community pharmacists (SCF-CP), which could serve to assess the competencies of community pharmacists in Lebanon and support their professional development and career progression.

## Methods

### Tool update and content validity

A team of experts gathered and reviewed the content of the previously suggested and validated community pharmacy framework, which included five domains [20]. The team of experts comprised four academics (two of whom are preceptors in community settings and two in clinical settings) and two community pharmacists.

After a thorough literature review, domains, competencies, and behaviors (items) were reviewed and adapted to the Lebanese setting. Additional behaviors were added, and the existing ones were adjusted based on several international studies and available frameworks [20–39]. A sixth domain describing “Preparedness and Response to Emergencies”, with behaviors taken from other frameworks [37–40], was added further to the pandemic and the multiple crises that hit Lebanon. Using a Delphi technique, the framework was circulated to the experts on more than 5 rounds until a consensus of more than 90% on all items was reached. Afterwards, the questionnaire was sent to a group of 11 community pharmacists from the OPL for a final review. Questions and minor inconsistencies were resolved by a final discussion.

The finalized framework was subsequently adapted to be administered to pharmacists through standardized online questionnaires.

### Study design

A cross-sectional study was carried out from March to October 2022 through an online questionnaire created on Google Forms for ease of distribution on social platforms (Facebook, Instagram, LinkedIn, and WhatsApp groups). The snowball technique was applied to reach pharmacists working in community settings across the five Lebanese governorates (Beirut, Beqaa, Mount Lebanon, South Lebanon, and North Lebanon). All community pharmacists living in Lebanon were eligible to participate.

### Ethical aspect

The Lebanese International University (LIU) School of Pharmacy Research and Ethics Committee approved the study protocol (2022RC-041-LIUSOP). This study was conducted following the ethical principles outlined in the Declaration of Helsinki. Explanations about the topic and the different aspects of the study were available in the introductory section of the questionnaire. Respondents gave written consent before proceeding to the survey. Anonymity and confidentiality were ensured across the entire data collection process.

### Sample size calculation

Two different methods were used to determine the minimum sample size required for the study. The first involved using the CDC Epi-info software to calculate the minimum sample needed to achieve a 3% error rate, with a 95% confidence interval, 5% alpha error, and 80% power. Based on the assumption that 90% of working pharmacists in the community setting would meet the specialized competencies and domains, a minimum of 350 participants was necessary. The second method used the G-Power software, version 3.0.10, to calculate the minimum sample size based on an effect size of 0.0526 and squared multiple correlations of 0.05 (*R*^2^ deviation from 0) related to the Omnibus test of multiple regression. The study allowed for 20 predictors to be included in the model with a 5% alpha error and 80% power, resulting in a minimum sample size of *n* = 415.

The targeted sample size was increased to 500 participants to account for potential missing data.

### Questionnaire and variables

The questionnaire was in English, as this language is commonly spoken by healthcare professionals in Lebanon, and comprised two sections. The first section collected information related to sociodemographic features and professional status. The second section consisted of the scale-based framework, which covered six domains, each comprising a set of competencies with their related behaviors (Additional file 1).

### Sociodemographic data

In this part, participants were asked about their general sociodemographic data, including age, gender, area of work, university of graduation, highest educational level, years of experience, the number of working hours per day, and the number of working days per week.

### Domains, competencies, and behaviors

The final framework comprised six domains related to 25 competencies. **Domain 0** included one set of competencies related to fundamental skills and comprised five competencies: Legal considerations, Product procurement and management, Compounding, Pharmacy Operation, Quality Improvement, and Pharmacy Automation. **Domain 1** (Safe and Rational use of medicines) comprised six competencies, i.e., Clinical skills, Medication Therapy Management, Compliance and Adherence, Problem-Solving/Referrals, Over-the-Counter Medications, and Pharmacovigilance. **Domain 2** (Pharmacy Management) covered two competencies, i.e., Functions and Managed care/Drug Coverage Policies. **Domain 3** (Professional Skills) encompassed seven competencies, i.e., Health Literacy, Patient Communication, Health professional Communication, Team Communication, Leadership Abilities and Personal Skills, Drug Information Skills, and Ethical Consideration. **Domain 4** (Public Health Fundamentals) included the competency of Clinical application of Public Health. **Domain 5** (Preparedness and Response to Emergency) included four competencies, all related to the emergency setting: emergency preparedness and response, operation management, patient care and population health interventions, and evaluation, research, and dissemination for impact and outcomes. Based on every competency, behaviors were suggested, and answers were adapted using a Likert scale (1 = Not confident at all to 5 = Very confident).

### Statistical analysis

The data were analyzed using SPSS software version 25. Firstly, a factor analysis using the principal component analysis (PCA) technique was conducted for the self-assessment of behaviors based on competencies and domains. The Kaiser–Meyer–Olkin (KMO) coefficient, Bartlett’s test for sphericity, and the total percentage of variance explained were reported for every analysis. For structural validity measures, Pearson correlation coefficients were calculated to assess the correlation of the domains within their respective competencies and their association with the other domains and the whole framework. Cronbach’s alpha values were also calculated for every competency to assess internal consistency (reliability).

Secondly, a descriptive analysis was performed using counts and percentages for categorical variables and means and standard deviations for continuous measures. For competencies and domains, standardized means over 100 were used for ease of comparison. The normality of continuous variables was checked using a visual inspection. In the bivariate analysis, the mean grade of each competency domain was then analyzed with the sociodemographic characteristics and working experiences of pharmacists using independent sample T-test (two groups comparison), one-way ANOVA (multiple groups comparison), and Pearson correlation (association between continuous variables).

Afterward, six multivariable linear regression models were performed, taking the mean of each competency domain as the dependent variable and the sociodemographic characteristics and working experiences with P values lower than 0.2 in the bivariate analysis as independent variables. The results were reported as an unadjusted beta with a 95% confidence interval. In all cases, the level of significance was set at *P* < 0.05.

## Results

### Description of the sociodemographic characteristics and work experience

A total of 512 community pharmacists were included in the study; their mean age was 35.35 years (± 10.77). The majority (91.6%) had a bachelor's degree in pharmacy, 54.3% were females, and 69.9% were English educated. The average number of years of experience was 10.10 (± 8.56), the majority (78.3%) of pharmacists practiced in a community setting only, the average number of working days per week was 5.93 (± 0.84), and the average number of working hours per day was 8.91 (± 3.09). Table 1 displays the complete sociodemographic characteristics and work experience of the participants.

**Table 1:** Sociodemographic characteristics and work experience of community pharmacists

Variable	Frequency (%)
Gender	
Male	234 (45.7)
Female	278 (54.3)
Level of education*	
BS Pharmacy	469 (91.6)
PharmD/DPharm	194 (37.9)
Masters	105 (20.5)
PhD	49 (9.6)
Other	
The highest degree related to your main field of work	
BS Pharmacy	286 (56.0)
PharmD/DPharm	140 (27.4)
Masters	48 (9.4)
PhD	37 (7.2)
Name of university graduated as a pharmacist	
Lebanese University (UL)	116 (22.7)
Saint Joseph University (USJ)	40 (7.8)
American University of Beirut (AUB)	7 (1.4)
Beirut Arab University (BAU)	97 (18.9)
Lebanese American University (LAU)	28 (5.5)
Lebanese International University (LIU)	169 (33.0)
Outside Lebanon	55 (10.7)
Name of university earning the highest degree from	
Lebanese University (UL)	120 (23.4)
Saint Joseph University (USJ)	41 (8.0)
American University of Beirut (AUB)	12 (2.3)
Beirut Arab University (BAU)	87 (17.0)
Lebanese American University (LAU)	33 (6.4)
Lebanese International University (LIU)	159 (31.1)
Outside Lebanon / other	57(11.1)
Language of pharmacy education	
French	129 (25.2)
English	358 (69.9)
Other	25 (4.9)
Work Location	
Beirut	165 (32.2)
Mount Lebanon	133 (26.0)
North Lebanon	83 (16.2)
South Lebanon	62 (12.1)
Beqaa	69 (13.5)
Number of patients received per day	
< 10	12 (2.3)
10–50	201 (39.3)
50–100	219 (42.8)
> 100	80 (15.6)
Owner of the pharmacy	
Yes	276 (43.9)
No	236 (46.1)
Another field of work	
I do not have another field of work	401 (78.3)
Academia (teaching); preceptor	44 (8.6)
Clinical pharmacy	37 (7.2)
Other	15 (2.9)
Research	8 (1.6)
Medical representative	6 (1.2)
Government hospital	1 (0.2)
	Mean ± SD
Age (years)	35.35 ± 10.77
Number of working days per week	5.93 ± 0.84
Number of working hours per day	8.91 ± 3.09
Years of experience as a community pharmacist	10.10 ± 8.56

### Validation of the framework

#### Content validity

The final validated framework included six domains, 25 competencies, and 135 behaviors. The first domain (Domain 0: Fundamental Skills) comprised 27 behaviors categorized into legal considerations; product procurement and management; compounding; pharmacy operation; quality improvement; and pharmacy automation. The second domain (Domain 1: Safe and Rational Use of Medicines) involved 28 behaviors divided into clinical skills; medication therapy management; compliance and adherence; problem-solving and referrals; over-the-counter medicines; and pharmacovigilance. The third domain (Domain 2: Pharmacy Management) covered 16 behaviors grouped under managed care/drug coverage policies. The fourth domain (Domain 3: Professional Skills) included 30 behaviors categorized into health literacy; patient communication; health professional communication; team communication; leadership abilities and personal skills; drug information skills; and ethical considerations. The fifth domain (Domain 4: Public Health Fundamentals) comprised 8 behaviors grouped under clinical applications of public health. Finally, the sixth domain (Domain 5 Emergency Preparedness and Response) included 26 behaviors categorized into emergency preparedness and response; operations management; patient care and population health interventions; and evaluation, research, and dissemination for impact and outcomes.

#### Factor analysis

Factor analysis was run to confirm the construct validity of the framework domains. All competencies could be extracted with Promax rotation and loaded appropriately on their domain. All competencies were loaded on one factor, and no variables had low factor loading (< 0.3), low communality (< 0.3), or over-correlation with each other (*r* > 0.9). The KMO measures of sampling adequacy were satisfactory for all models ranging from 0.500 to 0.956 with a significant Bartlett’s test of sphericity (*P* < 0.001). The percentage of explained variance ranged from 64.30 to 87.7%. Table 2 presents the Promax rotated matrix of factor analysis of the Lebanese community pharmacists’ competencies.

**Table 2:** Factor analysis (Promax rotated component matrix) and internal consistency of the framework

Domain/competency (sub-domain)	Behaviors	Loading	Cronbach alpha
Domain 0: Fundamental Skills			
0.1 Legal Considerations	0.1.2 Identify issues, pending legislation, and regulations across all levels of government	0.917	0.803
	0.1.1 Apply laws and regulations that impact pharmacy practice	0.917	
*Kaiser–Meyer–Olkin (KMO)* = *0.500; Bartlett’s test of sphericity P* < *0.001; Percentage of variance explained 84.08%*			
0.2 Product Procurement & Management	0.2.2 Anticipate, identify and troubleshoot problems with the supply chain	0.886	0.855
	0.2.3 Manage inventory	0.875	
	0.2.1 Select and acquire products through the appropriate supply chain	0.822	
	0.2.4 Handle drug waste	0.779	
*Kaiser–Meyer–Olkin (KMO)* = *0.784; Bartlett’s test of sphericity P* < *0.001; Percentage of variance explained 70.85%*			
0.3 Compounding	0.3.2 Perform elementary, non-sterile compounding: to be removed?	0.919	0.816
	0.3.1 Compound extemporaneous preparations	0.919	
*Kaiser–Meyer–Olkin (KMO)* = *0.500; Bartlett’s test of sphericity P* < *0.001; Percentage of variance explained 84.50%*			
0.4 Pharmacy Operation	0.4.3 Comprehend and adopt a given set of pharmacy operating procedures	0.851	0.953
	0.4.2 Apply typical pharmacy dispensing workflow	0.834	
	0.4.12 Work on the skill attitude and value that is essential to the practice and profession	0.832	
	0.4.9 Balance concrete specific motivation with constructive and professional criticism	0.826	
	0.4.8 Describe the roles and responsibilities of each pharmacy staff member	0.821	
	0.4.5 Evaluate prescription for legitimate medical use	0.820	
	0.4.4 Comprehend and adopt an existing collaborative drug therapy management system	0.819	
	0.4.10 Set and work towards meaningful goals with your staff	0.790	
	0.4.6 Prioritize and thoroughly complete tasks even with multiple interruptions occurring concurrently	0.777	
	0.4.13 Monitor the progress of the staff	0.774	
	0.4.7 Implement dispensing processes when pharmacy automation is utilized	0.762	
	0.4.11 Allow training development by staff and supervisors	0.761	
	0.4.1 Managing pharmacy operations efficiently	0.748	
*Kaiser–Meyer–Olkin (KMO)* = *0.956; Bartlett’s test of sphericity P* < *0.001; Percentage of variance explained 64.30%*			
0.5 Quality Improvement	0.5.3 Develop a plan for quality/performance improvement	0.897	0.848
	0.5.1 Optimize the concepts of quality measurement and improvement	0.875	
	0.5.2 Apply national/international standards/guidelines/best practices related to your community pharmacy practice	0.864	
*Kaiser–Meyer–Olkin (KMO)* = *0.726; Bartlett’s test of sphericity P* < *0.001; Percentage of variance explained 77.19%*			
0.6 Pharmacy Automation	0.6.1 Own a computerized system for dispensing medications	0.893	0.853
	0.6.2 Understand the role of computerized pharmacy management systems in dispensing	0.890	
	0.6.3 Dispense prescriptions utilizing technology-assisted workflow when applicable	0.858	
*Kaiser–Meyer–Olkin (KMO)* = *0.727; Bartlett’s test of sphericity P* < *0.001; Percentage of variance explained 77.46%*			
Domain 1: Safe and Rational Use of Medicines			
Clinical Skills	1.1.5 Demonstrate knowledge of appropriate administration techniques for dosage forms commonly dispensed in community pharmacies	0.872	0.948
	1.1.7 Proactively perform counseling and education which comply with current guidelines	0.871	
	1.1.6 Describe common doses of drugs requiring monitoring and collaborative drug therapy management	0.869	
	1.1.4 Describe and apply clinical practice guidelines to patient care	0.858	
	1.1.3 Individualize therapy through the implementation of a patient’s profile to the selection and modification of a medication regimen in collaboration with the prescriber	0.842	
	1.1.9 Commonly monitor for medication errors, drug interactions, and lab tests	0.838	
	1.1.2 Ensure the optimal use of medicines	0.833	
	1.1.8 Assist patients with chronic diseases regarding the appropriate use of chronic medications	0.816	
	1.1.1 Demonstrate and routinely apply clinical skills and provide patient care services	0.779	
*Kaiser–Meyer–Olkin (KMO)* = *0.949; Bartlett’s test of sphericity P* < *0.001; Percentage of variance explained 70.98%*			
1.2 Medication Therapy Management	1.2.5 Identify and resolve medication therapy problems, manage drug interactions, and resolve gaps in care	0.839	0.918
	1.2.4 Conduct a comprehensive medication review	0.825	
	1.2.3 Conduct a patient interview and provide education	0.808	
	1.2.7 Document services and follow-up with other health professionals	0.804	
	1.2.6 Recommend therapeutic alternatives and generic substitutions in collaboration with the prescriber	0.803	
	1.2.2 Define and appropriately document comprehensive MTM services	0.785	
	1.2.1 Develop a patient-centered, culturally responsive approach to medication management	0.779	
	1.2.8 Use multiple MTM platforms as required by third-party payers and OPL	0.771	
*Kaiser–Meyer–Olkin (KMO)* = *0.921; Bartlett’s test of sphericity P* < *0.001; Percentage of variance explained 64.32%*			
1.3 Compliance and Adherence	1.3.2 Identify and resolve patient-specific barriers to medication adherence	0.889	0.849
	1.3.1 Support and assist patient behavior change	0.878	
	1.3.3 Facilitate patient self-administration of medications and disease monitoring for minor diseases	0.863	
*Kaiser–Meyer–Olkin (KMO)* = *0.728; Bartlett’s test of sphericity P* < *0.001; Percentage of variance explained 76.83%*			
1.4 Problem-Solving/ Referrals	1.4.1 Make appropriate recommendations or referrals	0.936	0.860
	1.4.2 Assess and resolve issues related to medication safety	0.936	
*Kaiser–Meyer–Olkin (KMO)* = *0.500; Bartlett’s test of sphericity P* < *0.001; Percentage of variance explained 87.70%*			
1.5 Over-the-Counter Medicine	1.5.3 Assist with patient self-care, including helping patients make appropriate selections of herbal supplements	0.907	0.871
	1.5.2 Assist with patient self-care, including helping patients make appropriate selections of dietary supplements	0.898	
	1.5.1 Assist with patient self-care, including helping patients make appropriate selections of OTC medications	0.875	
*Kaiser–Meyer–Olkin (KMO)* = *0.736; Bartlett’s test of sphericity P* < *0.001; Percentage of variance explained 79.80%*			
1.6 Pharmacovigilance	1.6.3 Demonstrate knowledge of reporting an adverse drug reaction to relevant authorities	0.894	0.858
	1.6.2 Consider that reporting an ADR is part of pharmacist duties	0.888	
	1.6.1 Identify a potential adverse drug reaction	0.869	
*Kaiser–Meyer–Olkin (KMO)* = *0.733; Bartlett’s test of sphericity P* < *0.001; Percentage of variance explained 78.06%*			
Domain 2: Pharmacy Management			
2.1 Functions	2.1.4 Describe basic finance terms and analyze a financial statement	0.885	0.930
	2.1.3 Develop a business plan for clinical service programs	0.870	
	2.1.2 Identify cash flow problems and apply solutions to address them	0.859	
	2.1.6 Describe strategies for asset protection and safety	0.858	
	2.1.5 Apply healthcare economics and pharmacoeconomics	0.853	
	2.1.7 Use pharmacy technology effectively	0.783	
	2.1.1 Manage inventory costs and inventory levels or order points	0.762	
*Kaiser–Meyer–Olkin (KMO)* = *0.589; Bartlett’s test of sphericity P* < *0.001; Percentage of variance explained 70.51%*			
2.2 Managed Care/Drug Coverage Policies	2.2.7 Identify major factors influencing drug costs for a managed care organization (e.g., pharmacy costs, drug pricing methodologies, contracts/rebates, discounts)	0.872	0.936
	2.2.6 Identify major factors that contribute to prescription drug-related fraud and abuse	0.856	
	2.2.5 Discuss the concept of drug utilization review, and formulary management and provide functional definitions of key managed care strategies (e.g., prior authorizations, step therapy, quantity limits)	0.849	
	2.2.3 Provide guidance to patients seeking assistance to apply for drug payment programs	0.836	
	2.2.1 Explain the general concept of managed care, associated with the benefit structure of a health plan	0.792	
	2.2.8 Meet payer requirements for reimbursement	0.792	
	2.2.4 Troubleshoot denied claims	0.790	
	2.2.2 Adapt best treatment strategies to patient socioeconomic status	0.776	
	2.2.9 Reducing drug costs for the NSSF by providing less expensive drugs when applicable	0.770	
*Kaiser–Meyer–Olkin (KMO)* = *0.940; Bartlett’s test of sphericity P* < *0.001; Percentage of variance explained 66.53%*			
Domain 3: Professional Skills			
3.1 Health Literacy	3.1.2 Adjust counseling delivery and communicate at all levels of health literacy	0.890	0.851
	3.1.3 Solve adherence challenges created by low health literacy	0.886	
	3.1.1 Determine the patient's level of health literacy by observation or interview	0.860	
*Kaiser–Meyer–Olkin (KMO)* = *0.728; Bartlett’s test of sphericity P* < *0.001; Percentage of variance explained 77.25%*			
3.2 Patient Communication	3.2.4 Demonstrate respect for patient confidentiality and privacy rights	0.897	0.920
	3.2.3 Support patient behavior change through skills such as motivational interviewing	0.888	
	3.2.5 Demonstrate patient compassion and empathy	0.887	
	3.2.1 Listen closely and attentively to patients	0.849	
	3.2.2 Discuss pharmaceutical and other medical information thoroughly with patients and any family members	0.838	
*Kaiser–Meyer–Olkin (KMO)* = *0.876; Bartlett’s test of sphericity P* < *0.001; Percentage of variance explained 76.06%*			
3.3 Health Professional Communication	3.3.2 Contribute to effective interdisciplinary collaboration and integrated care	0.900	0.876
	3.3.1 Communicate effectively with colleagues, prescribers, and other health care providers	0.895	
	3.3.3 Document appropriate therapeutic recommendations related to medication therapy	0.893	
*Kaiser–Meyer–Olkin (KMO)* = *0.743; Bartlett’s test of sphericity P* < *0.001; Percentage of variance explained 80.28%*			
3.4 Team Communication	3.4.3 Supervise and motivate employees, staff, students, interns, and residents	0.883	0.908
	3.4.4 Delegate tasks appropriately	0.869	
	3.4.2 Identify and manage conflict at all levels	0.862	
	3.4.5 Articulate team objectives and measure and report team performance	0.849	
	3.4.1 Communicate with the pharmacy team, colleagues, prescribers, and other care providers in an efficient manner	0.817	
*Kaiser–Meyer–Olkin (KMO)* = *0.872; Bartlett’s test of sphericity P* < *0.001; Percentage of variance explained 73.33%*			
3.5 Leadership Abilities & Personal Skills	3.5.4 Be a gatekeeper to patient health	0.879	0.927
	3.5.5 Organize work and balance patient care and personal development	0.869	
	3.5.3 Embrace and advocate changes that improve patient care	0.851	
	3.5.6 Engage in regular professional development activities	0.824	
	3.5.7 Be active in professional organizations	0.817	
	3.5.2 Demonstrate professional behavior (attitude, dress, appearance, etc.) in practice settings	0.814	
	3.5.1 Display confidence in patient care skills	0.789	
*Kaiser–Meyer–Olkin (KMO)* = *0.916; Bartlett’s test of sphericity P* < *0.001; Percentage of variance explained 69.76%*			
3.6 Drug Information Skills	3.6.5 Deliver timely drug information to the general public and other health professionals	0.863	0.891
	3.6.2 Utilize a variety of drug-related reports, monographs, reviews, and policies using drug literature evaluation skills	0.851	
	3.6.3 Evaluate the appropriateness of clinical trials and other study designs, including validation of methodology and assessment of data credibility	0.846	
	3.6.6 Implement career advancement through continuous professional development	0.832	
	3.6.1 Access and utilize appropriate drug information resources and provide an accurate and credible solution in both written and oral forms	0.780	
*Kaiser–Meyer–Olkin (KMO)* = *0.847; Bartlett’s test of sphericity P* < *0.001; Percentage of variance explained 69.68%*			
3.7 Ethical Considerations	3.7.1 Understand professional ethics as they apply to the practice of pharmacy	0.933	0.850
	3.7.2 Apply knowledge and understanding of ethical aspects of pharmacy practice required to evaluate a patient care decision	0.933	
*Kaiser–Meyer–Olkin (KMO)* = *0.500; Bartlett’s test of sphericity P* < *0.001; Percentage of variance explained 87.04%*			
Domain 4: Public health Fundamentals			
4.1 Clinical Applications of Public Health	4.1.5 Collect, assess, and make recommendations based on the results of health and wellness screenings and diagnostic tests	0.887	0.939
	4.1.6 Promote healthy lifestyle and nutrition and describe how it impacts drug therapy and overall health/well-being	0.863	
	4.1.4 Be knowledgeable about immunization schedules and requirements and actively involved in vaccination campaigns	0.856	
	4.1.7 Describe the role of a pharmacist in emergency situations	0.850	
	4.1.8 Participate in the population-based provision of care (as distinguished from direct patient care)	0.844	
	4.1.3 Participate in education and intervention in public health initiatives applicable to pharmacy practice	0.834	
	4.1.2 Educate population to access and understand health information on the selection and rational use of medicines and other health products	0.813	
	4.1.1 Assess and support local and national health priorities and initiatives	0.756	
*Kaiser–Meyer–Olkin (KMO)* = *0.934; Bartlett’s test of sphericity P* < *0.001; Percentage of variance explained 70.34%*			
Domain 5: Emergency Preparedness and Response (EPR)			
5.1 Emergency Preparedness and Response	5.1.5 Partner with local authorities	0.868	0.933
	5.1.3 Address medication shortage and mitigation plan	0.862	
	5.1.4 Balance stockpile and availability of drugs for existing/chronic conditions	0.828	
	5.1.6 Check for FDA/EMA Emergency Use Authorizations (EUAs) and expedited review and approval of tests/drugs for treatment	0.823	
	5.1.2 Check for training opportunities	0.822	
	5.1.1 Check for volunteering opportunities	0.803	
	5.1.8 Involve trainees and staff in emergency response	0.801	
	5.1.7 Follow actions and recommendations of local authorities	0.797	
*Kaiser–Meyer–Olkin (KMO)* = *0.902; Bartlett’s test of sphericity P* < *0.001; Percentage of variance explained 68.21%*			
5.2 Operations Management	5.2.3 Develop workplace training and safety protocols (e.g., social distancing)	0.881	0.933
	5.2.6 Adapt working hours to meet essential services during crises	0.866	
	5.2.5 Monitor workers/assistants for symptoms	0.859	
	5.2.7 Secure sanitizers and other medications when needed	0.855	
	5.2.4 Secure PPEs or other needed materials	0.852	
	5.2.2 Ensure medication delivery/safe storage	0.851	
	5.2.1 Procure essential medications and supplies	0.799	
	5.2.8 Participate in interdisciplinary training for EPR teams	0.699	
*Kaiser–Meyer–Olkin (KMO)* = *0.935; Bartlett’s test of sphericity P* < *0.001; Percentage of variance explained 69.62%*			
5.3 Patient Care and Population Health Interventions	5.3.4 Educate the patient about the ongoing crisis using evidence-based information and communications	0.851	0.893
	5.3.3 Identify at-risk populations	0.839	
	5.3.2 Continue medication reviews, screening, and/or testing/vaccination services safely	0.829	
	5.3.5 Manage panic buying	0.799	
	5.3.1 Maintain patient confidentiality	0.782	
	5.3.6 Answer EPR-related calls	0.751	
*Kaiser–Meyer–Olkin (KMO)* = *0.886; Bartlett’s test of sphericity P* < *0.001; Percentage of variance explained 65.51%*			
5.4 Evaluation, Research, and Dissemination for Impact and Outcomes	5.4.2 Publish and/or disseminate findings	0.933	0.926
	5.4.1 Participate in research and studies on EPR	0.910	
	5.4.3 Combat misinformation by disseminating evidence-based information to patients and sharing it on social media	0.892	
	5.4.4 Develop training programs for peers and other healthcare workers	0.885	
*Kaiser–Meyer–Olkin (KMO)* = *0.839; Bartlett’s test of sphericity P* < *0.001; Percentage of variance explained 81.95%*			

#### Validity measures

The internal consistency of all competencies and domains was confirmed by measuring Cronbach’s alpha values, which ranged from very good (0.803) to excellent (0.953).

The values varied as follows: Domain 0 from 0.803 to 0.953, Domain 1 from 0.849 to 0.948, Domain 2 from 0.930 to 0.936, Domain 3 from 0.850 to 0.927, Domain 4 from 0.939, and Domain 5 from 0.893 to 0.933 (Table 2). Furthermore, all competencies were significantly correlated with their respective domains, with all values of *P* < 0.001, and there was a significant correlation between all domains and between each domain and the total framework (*P* < 0.001). The Pearson correlation analysis is shown in Table 3.

**Table 3:** Structural validity through Pearson correlation analysis of the framework domains

	Domain 0	Domain 1	Domain 2	Domain 3	Domain 4	Domain 5
Total framework	0.902	0.942	0.879	0.948	0.881	0.917
Domain 0		0.840	0.737	0.802	0.751	0.771
0.1 Legal Considerations	0.711					
0.2 Product Procurement and Management	0.839					
0.3 Compounding	0.547					
0.4 Pharmacy Operation	0.945					
0.5 Quality Improvement	0.740					
0.6 Pharmacy Automation	0.704					
Domain 1	0.840		0.790	0.881	0.788	0.814
1.1 Clinical Skills		0.913				
1.2 Medication Therapy Management		0.903				
1.3 Compliance and Adherence		0.845				
1.4 Problem-Solving/ Referrals		0.808				
1.5 Over-the-Counter Medicine		0.812				
1.6 Pharmacovigilance		0.779				
Domain 2	0.737	0.790		0.818	0.752	0.774
2.1 Functions			0.928			
2.2 Managed Care/Drug Coverage Policies			0.948			
Domain 3	0.802	0.881	0.818		0.837	0.832
3.1 Health Literacy				0.831		
3.2 Patient Communication				0.861		
3.3 Health Professional Communication				0.847		
3.4 Team Communication				0.918		
3.5 Leadership Abilities and Personal Skills				0.929		
3.6 Drug Information Skills				0.847		
3.7 Ethical Considerations				0.822		
Domain 4	0.751	0.788	0.752	0.837		0.829
Domain 5	0.771	0.814	0.774	0.832	0.829	
5.1 Emergency Preparedness and Response						0.887
5.2 Operations Management						0.891
5.3 Patient Care and Population Health Interventions						0.854
5.4 Evaluation, Research, and Dissemination for Impact and Outcomes						0.751

*All computed *p*-values are < 0.001

Domain 0 = Fundamental Skills; Domain 1 = Safe and Rational Use of medicines; Domain 2 = Pharmacy Management; Domain 3 = Professional Skills; Domain 4 = Public Health Fundamentals; Domain 5 = Preparedness and Response to Emergency

#### Assessment of competencies

**Fig. 1:**
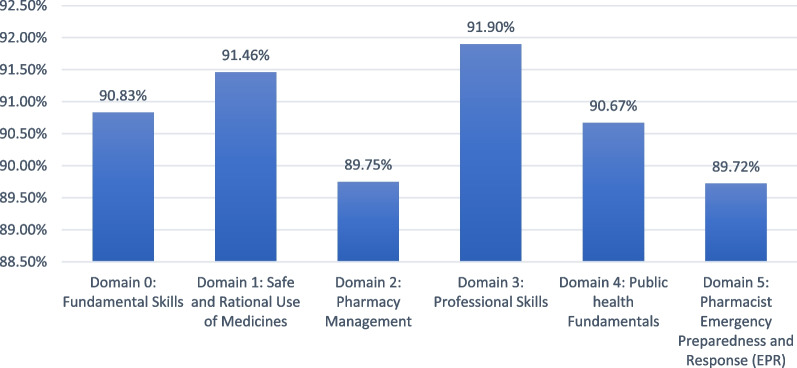
Assessment of the 6 domains of the framework among community pharmacists

Figure 1 presents the assessment of the 6 domains of the framework among community pharmacists. Participants declared being competent in all domains, with a percentage of mean grade ranging from 90.67% for public health fundamentals to 91.90% for professional skills. Two domains had slightly lower grades, i.e., 89.75% for pharmacy management and 89.72% for emergency preparedness and response.

The assessment of each competency within the domains showed that the competencies with the lowest means were compounding (82.3%; fundamental skills), medication therapy management (90.1%; safe and rational use of medicines), functions (89.5%; pharmacy management), drug information skills (90.6%; professional skills), and emergency preparedness and response (88.7%). Table 4 presents a detailed assessment of the domains and competencies.

**Table 4:** Assessment of the framework domains and competencies

	Mean ± SD	% of mean	Median	Minimum	Maximum
Domain 0: Fundamental Skills	122.63 ± 13.30	90.83	127.00	54.00	135.00
0.1 Legal Considerations	9.18 ± 1.26	91.8	10.00	2.00	10.00
0.2 Product Procurement and Management	18.26 ± 2.27	91.3	19.00	8.00	20.00
0.3 Compounding	8.23 ± 1.93	82.3	8.00	2.00	10.00
0.4 Pharmacy Operation	59.73 ± 7.04	91.89	62.50	26.00	65.00
0.5 Quality Improvement	13.60 ± 1.91	90.66	14.00	3.00	15.00
0.6 Pharmacy Automation	13.61 ± 1.94	90.73	15.00	5.00	15.00
Domain 1: Safe and Rational Use of Medicines	128.05 ± 14.18	91.46	133.00	56.00	140.00
1.1 Clinical Skills	41.31 ± 5.31	91.8	44.00	16.00	45.00
1.2 Medication Therapy Management	36.03 ± 4.66	90.07	37.00	16.00	40.00
1.3 Compliance and Adherence	13.85 ± 1.62	92.33	15.00	6.00	15.00
1.4 Problem-Solving/ Referrals	9.30 ± 1.12	93.0	10.00	4.00	10.00
1.5 Over-the-Counter Medicine	13.96 ± 1.65	93.06	15.00	6.00	15.00
1.6 Pharmacovigilance	13.57 ± 1.91	90.46	14.00	3.00	15.00
Domain 2: Pharmacy Management	71.80 ± 9.28	89.75	74.00	27.00	80.00
2.1 Functions	31.34 ± 4.56	89.54	33.00	7.00	35.00
2.2 Managed Care/Drug Coverage Policies	40.64 ± 5.33	90.31	42.00	18.00	45.00
Domain 3: Professional Skills	137.85 ± 15.06	91.9	143.00	60.00	150.00
3.1 Health Literacy	13.64 ± 1.84	90.93	15.00	6.00	15.00
3.2 Patient Communication	23.21 ± 2.79	92.84	25.00	7.00	25.00
3.3 Health Professional Communication	13.88 ± 1.70	92.53	15.00	6.00	15.00
3.4 Team Communication	22.96 ± 2.81	91.84	25.00	10.00	25.00
3.5 Leadership Abilities and Personal Skills	32.15 ± 3.96	91.85	34.00	14.00	35.00
3.6 Drug Information Skills	22.64 ± 3.00	90.56	24.00	10.00	25.00
3.7 Ethical Considerations	9.35 ± 1.06	93.5	10.00	4.00	10.00
Domain 4: Public health Fundamentals					
4.1 Clinical Applications of Public Health	36.27 ± 4.91	90.67	38.00	13.00	40.00
Domain 5: Emergency Preparedness and Response (EPR)	116.64 ± 14.05	89.72	120.00	52.00	130.00
5.1 Emergency Preparedness and Response	35.48 ± 5.21	88.70	37.00	14.00	40.00
5.2 Operations Management	36.59 ± 4.60	91.47	39.00	16.00	40.00
5.3 Patient Care and Population Health Interventions	27.49 ± 3.24	91.63	29.00	12.00	30.00
5.4 Evaluation, Research, and Dissemination for Impact and Outcomes	17.07 ± 3.41	85.35	18.00	4.00	20.00

### Bivariate analysis

In the bivariate analysis, higher means of the fundamental skills domain were significantly associated with being a pharmacy owner (*P* = 0.020), not having another field (non-community) of work (*P* = 0.017), older age (*P* < 0.001), a higher number of working days per week (P = 0.018), and more years of experience as a community pharmacist (*P* < 0.001). Fundamental skills were also significantly different, according to work locations (*P* = 0.001) and the number of patients received per day (*P* < 0.001).

Higher means of safe and rational use of medicines were significantly associated with being a pharmacy owner (*P* = 0.009), not having another field (non-community) of work (*P* = 0.005), older age (*P* < 0.001), a higher number of working days per week (*P* = 0.011), and more years of experience as a community pharmacist (*P* < 0.001). Safe and rational use of medicines were also significantly different, according to the language of education (*P* = 0.020), work location (*P* < 0.020), and the number of patients received per day (*P* = 0.001).

Higher means of pharmacy management were significantly associated with being a male pharmacist (*P* = 0.015), a pharmacy owner (*P* = 0.002), not having another field (non-community) of work (*P* = 0.036), older age (*P* = 0.002), a higher number of working days per week (*P* = 0.001), and more years of experience as a community pharmacist (P = 0.001). Pharmacy management was also significantly different, according to work location (*P* = 0.004) and the number of patients received per day (*P* = 0.026).

Higher means of professional skills domain were significantly associated with older age (*P* < 0.001), a higher number of working days per week (*P* = 0.008), not with having another field (non-community) of work (*P* = 0.011), and more years of experience as a community pharmacist (*P* < 0.001). Professional skills were also significantly different according to work location (*P* = 0.001), and the number of patients received per day (*P* = 0.004).

Higher means of public health fundamentals were significantly associated with older age (*P* = 0.006), a higher number of working days per week (*P* = 0.009), and more years of experience as a community pharmacist (*P* = 0.007). Public health fundamentals were also significantly different according to the number of patients received per day (*P* = 0.003).

Higher means of emergency preparedness and response were significantly associated with being a male pharmacist (*P* = 0.005), a pharmacy owner (*P* = 0.026), not having another field (non-community) of work (*P* = 0.038), older age (*P* = 0.006), a higher number of working days per week (*P* = 0.040), and more years of experience as a community pharmacist (*P* = 0.003). Emergency preparedness and response were also significantly different, according to work location (*P* = 0.038) and the number of patients received per day (*P* = 0.008).

The bivariate analysis taking the community pharmacists' competency domains as the dependent variables is shown in Table 5.

**Table 5:** Bivariate analysis taking the framework domains as the dependent variables

	Domain 0—Fundamental Skills	Domain 1—Safe and Rational Use of Medicines	Domain 2—Pharmacy Management	Domain 3—Professional Skills	Domain 4—Public health Fundamentals	Domain 5—Emergency Preparedness and Response (EPR)
Gender						
Male	123.41 ± 13.37	129.11 ± 13.68	72.88 ± 8.85	138.50 ± 14.63	36.70 ± 4.71	118.53 ± 12.85
Female	121.98 ± 13.22	127.16 ± 14.56	70.89 ± 9.54	137.30 ± 15.42	35.90 ± 5.05	115.04 ± 14.83
* p-value*	0.226	0.123	0.015	0.370	0.065	0.005
Language of pharmacy education						
French	122.89 ± 12.85	128.45 ± 13.03	71.17 ± 9.78	137.72 ± 13.73	35.88 ± 5.07	116.77 ± 13.59
English	122.19 ± 13.69	127.39 ± 14.78	71.76 ± 9.24	137.41 ± 15.80	36.25 ± 4.94	116.22 ± 14.25
Other	127.72 ± 8.08	135.52 ± 7.87	75.72 ± 6.01	144.80 ± 7.75	38.48 ± 2.72	121.88 ± 13.04
* p-value*	0.129	0.020	0.080	0.060	0.053	0.150
Work Location						
Beirut	119.52 ± 15.47	124.15 ± 16.69	70.01 ± 10.09	134.00 ± 17.55	35.79 ± 5.11	114.42 ± 14.67
Mount Lebanon	124.46 ± 10.02	129.99 ± 11.55	71.39 ± 9.92	140.28 ± 12.16	36.12 ± 5.21	117.09 ± 14.04
North Lebanon	122.85 ± 13.49	128.63 ± 13.76	72.67 ± 8.37	138.50 ± 13.98	36.57 ± 4.41	117.44 ± 13.68
South Lebanon	127.20 ± 11.16	132.14 ± 12.56	74.87 ± 7.06	141.90 ± 14.24	37.48 ± 4.20	120.83 ± 12.63
Beqaa	122.20 ± 13.27	129.27 ± 12.23	73.08 ± 7.86	137.97 ± 13.94	36.26 ± 4.90	116.31 ± 13.57
* p-value*	0.001	< 0.001	0.004	0.001	0.216	0.038
Number of patients received per day						
< 10	111.33 ± 27.34	116.66 ± 27.30	66.41 ± 14.44	125.33 ± 29.07	32.83 ± 7.67	109.58 ± 23.42
10—50	120.76 ± 13.35	126.12 ± 14.70	70.81 ± 9.00	136.43 ± 15.44	35.60 ± 5.23	114.66 ± 14.54
50—100	123.84 ± 12.71	129.46 ± 13.42	72.82 ± 8.95	139.06 ± 14.18	36.77 ± 4.57	117.77 ± 13.62
> 100	125.73 ± 10.04	130.76 ± 10.55	72.32 ± 9.57	140.00 ± 12.41	37.10 ± 4.03	119.55 ± 11.15
* p-value*	< 0.001	0.001	0.026	0.004	0.003	0.008
Owner of the pharmacy						
Yes	123.90 ± 13.21	129.57 ± 13.77	73.00 ± 8.72	138.95 ± 14.61	36.56 ± 4.90	117.91 ± 13.51
No	121.16 ± 13.28	126.27 ± 14.48	70.40 ± 9.73	136.57 ± 15.51	35.93 ± 4.91	115.14 ± 14.56
* p-value*	0.020	0.009	0.002	0.075	0.151	0.026
Another field of work						
I do not have another field of work	123.45 ± 12.69	129.11 ± 13.30	72.32 ± 8.67	138.90 ± 13.85	36.46 ± 4.74	117.31 ± 13.58
I have another work	119.69 ± 15.00	124.22 ± 16.51	69.92 ± 11.06	134.07 ± 18.38	35.59 ± 5.45	114.19 ± 15.48
* p-value*	0.017	0.005	0.036	0.011	0.130	0.038
	Correlation coefficient	Correlation coefficient	Correlation coefficient	Correlation coefficient	Correlation coefficient	Correlation coefficient
Age	0.180	0.170	0.140	0.160	0.122	0.121
*p-value*	< 0.001	< 0.001	0.002	< 0.001	0.006	0.006
Number of working days per week	0.104	0.112	0.144	0.117	0.116	0.091
*p-value*	0.018	0.011	0.001	0.008	0.009	0.040
Number of working hours per day	0.073	0.076	0.068	0.048	0.077	0.084
*p-value*	0.097	0.084	0.125	0.276	0.082	0.057
Years of experience as a community pharmacist	0.211	0.182	0.146	0.181	0.119	0.130
*p-value*	< 0.001	< 0.001	0.001	< 0.001	0.007	0.003

### Multivariable analysis

Six models of multivariable linear regression, taking the framework domains as the dependent variables, were performed.

Better fundamental skills were significantly associated with more years of experience as a community pharmacist (Beta = 0.281, *P* = 0.001) and receiving more than 50 patients per day (Beta = 3.389, *P* = 0.004) compared to receiving less than 50 patients per day. However, lower fundamental skills were significantly associated with having another field (non-community) of work (Beta = -3.022, *P* = 0.032).

Safer and more rational use of medicines was significantly associated with more years of practice experience as a community pharmacist (Beta = 0.184, *P* = 0.046) and receiving more than 50 patients per day (Beta = 3.508, *P* = 0.006) compared to receiving less than 50 patients per day. Lesser safe and rational use of medicines was significantly associated with having another field (non-community) of work (Beta = − 4.124, *P* = 0.006).

The pharmacy management competency domain was also significantly associated with receiving more than 50 patients per day (Beta = 1.699, *P* = 0.042) compared to receiving less than 50 patients per day, while it was significantly lower with having another field (non-community) of work (Beta = − 2.053, P = 0.038).

Better professional skills were significantly associated with more years of experience as a community pharmacist (Beta = 0.253, *P* = 0.010) and receiving more than 50 patients per day (Beta = 2.763, *P* = 0.041) compared to receiving less than 50 patients per day. Lower professional skills were significantly associated with having another field (non-community) of work (Beta = − 4.145, *P* = 0.010).

Better public health fundamentals were significantly associated with other languages of pharmacy education vs. the French language (Beta = 2.397, *P* = 0.034) and receiving more than 50 patients per day (Beta = 1.237, *P* = 0.005) compared to receiving less than 50 patients per day.

Finally, better emergency preparedness and response were significantly associated with receiving more than 50 patients per day (Beta = 3.188, *P* = 0.012) compared to receiving less than 50 patients per day.

The six models of multivariable linear regressions, taking the framework domains as the dependent variables, are shown in Table 6.

**Table 6:** Multivariable linear regressions taking the framework domains as the dependent variables

	Unstandardized beta	Standardized beta	*P*-value	Confidence interval	
				Lower bound	Upper bound
Model 1: Domain 0—Fundamental Skills					
Gender (Female vs Male*)	− 0.314	− 0.012	0.798	− 2.721	2.093
Number of working days per week	0.691	0.044	0.346	− 0.748	2.130
Number of working hours per day	0.001	0.0003	0.994	− 0.395	0.398
Owner of the pharmacy (Yes vs No*)	− 0.720	− 0.027	0.611	− 3.500	2.060
Another work (Yes vs I do not work*)	− 3.022	− 0.094	0.032	− 5.788	− 0.255
Years of experience as a community pharmacist	0.281	0.181	0.001	0.112	0.450
Language of pharmacy education (English vs French*)	1.129	0.039	0.481	− 2.014	4.271
Language of pharmacy education (Other vs French*)	2.859	0.046	0.345	− 3.081	8.800
Number of patients (Over 50 vs under 50*)	3.389	0.126	0.004	1.059	5.719
Name of university graduated as a pharmacist (UL vs other*)	− 0.017	− 0.001	0.992	− 3.251	3.217
Name of university graduated as a pharmacist (LIU vs Other*)	− 0.400	− 0.014	0.790	− 3.349	2.548
Model 2: Domain 1—Safe and Rational Use of Medicines					
Gender (Female vs Male*)	− 0.784	− 0.028	0.549	− 3.352	1.783
Number of working days per week	0.945	0.056	0.227	− 0.590	2.480
Number of working hours per day	0.010	0.002	0.962	− 0.413	0.433
Owner of the pharmacy (Yes vs No*)	− 0.009	0.000	0.995	− 2.974	2.956
Another work (Yes vs I do not work*)	− 4.124	− 0.120	0.006	− 7.075	− 1.173
Years of experience as a community pharmacist	0.184	0.111	0.046	0.004	0.364
Language of pharmacy education (English vs French*)	0.554	0.018	0.746	− 2.799	3.906
Language of pharmacy education (Other vs French*)	5.467	0.083	0.091	− 0.870	11.803
Number of patients (Over 50 vs under 50*)	3.508	0.122	0.006	1.023	5.994
Name of university graduated as a pharmacist (UL vs other*)	0.166	0.005	0.925	− 3.284	3.615
Name of university graduated as a pharmacist (LIU vs Other*)	− 0.506	− 0.017	0.752	− 3.651	2.640
Model 3: Domain 2—Pharmacy Management					
Gender (Female vs Male*)	− 1.097	− 0.059	0.203	− 2.786	0.593
Number of working days per week	0.938	0.086	0.069	− 0.072	1.948
Number of working hours per day	− 0.052	− 0.017	0.716	− 0.330	0.227
Owner of the pharmacy (Yes vs No*)	1.044	0.056	0.294	− 0.907	2.995
Another work (Yes vs I do not work*)	− 2.053	− 0.091	0.038	− 3.995	− 0.111
Years of experience as a community pharmacist	0.084	0.078	0.162	− 0.034	0.203
Language of pharmacy education (English vs French*)	1.456	0.072	0.195	− 0.749	3.662
Language of pharmacy education (Other vs French*)	3.794	0.088	0.074	− 0.376	7.964
Number of patients (Over 50 vs under 50*)	1.699	0.090	0.042	0.064	3.335
Name of university graduated as a pharmacist (UL vs other*)	0.741	0.033	0.522	− 1.529	3.011
Name of university graduated as a pharmacist (LIU vs Other*)	0.423	0.021	0.688	− 1.646	2.493
Model 4: Domain 3—Professional Skills					
Gender (Female vs Male*)	− 0.335	− 0.011	0.810	− 3.073	2.403
Number of working days per week	1.291	0.073	0.122	− 0.345	2.928
Number of working hours per day	− 0.085	− 0.017	0.712	− 0.535	0.366
Owner of the pharmacy (Yes vs No*)	− 1.475	− 0.049	0.360	− 4.637	1.687
Another work (Yes vs I do not work*)	− 4.145	− 0.113	0.010	− 7.292	− 0.998
Years of experience as a community pharmacist	0.253	0.144	0.010	0.061	0.445
Language of pharmacy education (English vs French*)	1.628	0.050	0.371	− 1.946	5.202
Language of pharmacy education (Other vs French*)	5.087	0.073	0.140	− 1.670	11.844
Number of patients (Over 50 vs under 50*)	2.763	0.090	0.041	0.113	5.413
Name of university graduated as a pharmacist (UL vs other*)	− 0.021	− 0.001	0.991	− 3.699	3.658
Name of university graduated as a pharmacist (LIU vs Other*)	− 1.613	− 0.050	0.345	− 4.966	1.741
Model 5: Domain 4—Public health Fundamentals					
Gender (Female vs Male*)	− 0.447	− 0.045	0.329	− 1.346	0.452
Number of working days per week	0.389	0.067	0.155	− 0.148	0.927
Number of working hours per day	0.033	0.021	0.665	− 0.115	0.181
Owner of the pharmacy (Yes vs No*)	− 0.358	− 0.036	0.498	− 1.396	0.680
Another work (Yes vs I do not work*)	− 0.739	− 0.062	0.161	− 1.771	0.294
Years of experience as a community pharmacist	0.038	0.066	0.236	− 0.025	0.101
Language of pharmacy education (English vs French*)	0.902	0.084	0.132	− 0.271	2.075
Language of pharmacy education (Other vs French*)	2.397	0.105	0.034	0.180	4.615
Number of patients (Over 50 vs under 50*)	1.237	0.124	0.005	0.367	2.107
Name of university graduated as a pharmacist (UL vs other*)	0.259	0.022	0.674	− 0.948	1.466
Name of university graduated as a pharmacist (LIU vs Other*)	− 0.452	− 0.043	0.420	− 1.553	0.649
Model 6: Domain 5—Emergency Preparedness and Response (EPR)					
Gender (Female vs Male*)	− 2.544	− 0.090	0.053	− 5.118	0.030
Number of working days per week	0.597	0.036	0.446	− 0.942	2.136
Number of working hours per day	0.077	0.017	0.721	− 0.347	0.501
Owner of the pharmacy (Yes vs No*)	0.077	0.003	0.959	− 2.896	3.050
Another work (Yes vs I do not work*)	− 2.649	− 0.078	0.079	− 5.608	0.309
Years of experience as a community pharmacist	0.102	0.062	0.269	− 0.079	0.282
Language of pharmacy education (English vs French*)	0.571	0.019	0.739	− 2.790	3.931
Language of pharmacy education (Other vs French*)	4.076	0.063	0.208	− 2.277	10.429
Number of patients (Over 50 vs under 50*)	3.188	0.112	0.012	0.696	5.680
Name of university graduated as a pharmacist (UL vs other*)	0.157	0.005	0.929	− 3.301	3.616
Name of university graduated as a pharmacist (LIU vs Other*)	− 0.445	− 0.015	0.781	− 3.599	2.708

Variables entered: gender, number of working days per week, number of working hours per day, owner of the pharmacy, another work, years of experience as a community pharmacy, language of pharmacy education, number of patients, name of university graduated as a pharmacist

*Reference group

## Discussion

This study could update and validate the first version of the Lebanese specialized competency framework for community pharmacy practitioners in conformity with the published literature. The six domains described comprised fundamental skills, safe and rational use of medicines, pharmacy management, professional skills, public health fundamentals, and emergency preparedness and response. The total number of competencies was 25, while the total number of behaviors was 135.

The necessity of a country-specific framework to assess pharmacists’ performance and considerations on how to enhance this framework was recognized by several countries around the world, although mainly applied to core competencies. Several studies from Australia [41], Croatia [42, 43], Ireland [44], Kuwait [14], Japan [45], New Zealand [21], Serbia [46], Thailand [47, 48], and 14 African countries, including Ghana, Kenya, Nigeria, and South Africa [49] were conducted to develop or translate and cross-culturally adapt a national competency framework to assess essential standardized recommendations implementation to advance local practice.

The notable feature of this framework consists of adapting it to the multifaceted crisis context of Lebanon [50] by including the emergency preparedness and response domain, which was not in the previously suggested framework. Emergency preparedness and response have become an emerging role of pharmacists, a rising area of pharmacy practice [51], and a significant concern given the recent global spread of COVID-19 [52]. According to the FIP, pharmacists have roles and responsibilities at all levels of emergency preparedness linked to functioning at individual and community levels [53]. Adapting the roles of the pharmacist in response to the needs of the public requires continuing development of pharmacists’ competencies to adequately participate in areas such as emergency response and disasters.

To the best of our knowledge, no previous studies have evaluated the construct validity and reliability of specialized competencies required by community pharmacists. Our findings demonstrated the construct validity of the Lebanese specialized competency framework for community pharmacists, showing that all competencies have high factor loading and thus confirming the adequacy of all the domains of the framework. The structural validity of the current framework was also confirmed by the highly significant correlation of each competency with its domain and the highly significant correlation of all domains with each other and with the entire framework. Analysis of additional psychometric properties of the framework verified its reliability, as indicated by the Cronbach alpha values for all competencies [54]. Thus, this framework is recommended for assessment in practice and research settings. Nevertheless, further validation measures such as criterion validity and test–retest reliability are still required, in addition to comparative studies with the competencies of other practice settings such as hospital and ambulatory care.

The present study also assessed the grade of each competency and domain among participants and evaluated the role of sociodemographic characteristics and work experience in predicting the competencies of community pharmacists. Our findings showed that community pharmacists appeared to be competent in all six domains. Yet, participants were found to be slightly less skilled in pharmacy management and emergency preparedness and response compared to other areas. The reason for the lower declared competence in pharmacy management could be attributed to the pharmacy education in Lebanon and the knowledge and skills acquired through the curricula. Indeed, pharmacy management is usually introduced in one or more didactic courses throughout the pharmacy programs, although it is not adequately enforced in practical experiences and entrustable professional activities [55, 56]. Worse grades in pharmacy management and other domains are expected soon due to the large number of fresh graduates who have undertaken their didactic courses remotely and practical experiences virtually over a considerable period during the COVID-19 lockdowns [57]. On the other hand, the low grades in emergency preparedness and readiness were not surprising as this is a new emerging role of pharmacists, and this domain is still relatively recent among the internationally recommended competencies [53]. Meanwhile, Lebanese pharmacists reportedly demonstrated high professional skills and expertise in providing community services and patient care amid the COVID-19 pandemic [58].

The present findings showed that better competencies are significantly associated with more years of practice experience, receiving more patients per day, and being a non-French educated pharmacist. However, lower competencies were significantly associated with having additional work outside the community setting. The domains of fundamental skills, safe and rational use of medicines, pharmacy management, professional skills, public health fundamentals, and emergency preparedness and response were declared to be significantly better among pharmacists who receive more than 50 patients per day. Further, the domains of fundamental skills, safe and rational use of medicines, and professional skills were significantly better among pharmacists with more years of work experience. Receiving a higher number of patients and having more years of practice correlated with better competence in several areas of community service provision and patient care. Although this association was not comprehensively explored previously, the literature reported that more work experience was associated with better knowledge and skills [59]. Of note, the degree of education was not linked to better-declared competencies, which reveals the gap between education and practice.

Public health fundamental skills were significantly higher among community pharmacists who received their education in languages other than French. The reason for this finding is not fully understood, but it could be due to the rating scheme that is consistently higher in the American versus the French system to which pharmacists have been exposed as students; the same was also found for core competencies assessment [18, 19]. It could also be that universities with French curricula do not focus on public health aspects of pharmacy. Further studies in this context are suggested to elucidate this association.

Moreover, pharmacists who got an additional non-community pharmacy work reported significantly lower competencies in the domains of fundamental skills, safe and rational use of medicines, pharmacy management, and professional skills. This result could be attributed to fatigue and less attentive practice in each work setting [60]. Lately, many Lebanese pharmacists have been obliged to secure multiple jobs to improve their financial situation during the current severe socioeconomic crisis affecting Lebanon. This reality was associated with longer working hours and lower proficiency [61].

### Implications for practice

The Lebanese specialized competency framework for community pharmacists carries significant implications for policy and practice globally and locally. Firstly, the framework serves to confirm the existing competencies that community pharmacists should possess while also identifying new emerging roles and areas for professional development. Thus, it provides a clear roadmap for pharmacists to enhance the quality of community services and patient care. This competency-based approach is crucial in an ever-evolving healthcare landscape where pharmacists play a vital role in ensuring safe and effective medication use. Moreover, this framework would potentially transform pharmacy practice, education, and continuing professional development by providing a structured approach to guide the education and training of future pharmacists and ensuring that they acquire the necessary skills and knowledge aligned with the evolving needs of the profession. It would also support ongoing professional development, enabling practicing pharmacists to continuously update their competencies and stay abreast of advancements in their field.

Another notable implication of this framework is its potential to establish standardized community pharmacy practice. Indeed, it creates a benchmark for professional practice and encourages consistency in service delivery across different pharmacy settings by defining the competencies expected from community pharmacists. This standardization promotes quality assurance and fosters public trust in the pharmacy profession. Furthermore, the framework has the potential to bridge the gap between different generations of pharmacists. Younger pharmacists may possess more updated theoretical knowledge, while older pharmacists bring valuable practical experiences. Both generations can benefit from a shared understanding of the necessary competencies by embracing this competency framework, thus facilitating collaboration and knowledge exchange for the benefit of patient care.

While the framework presents promising opportunities, it is essential to acknowledge the potential challenges its implementation might encounter. The unstable political situation in Lebanon might hinder the adoption of new policies and legislation, making it crucial for relevant authorities and academia to exert additional efforts. Continuous advocacy, engagement, and collaboration between policymakers, academia, and relevant stakeholders are necessary to overcome these obstacles and ensure the integration of these competencies into community pharmacy practice.

## Limitations and strengths

This study has several strengths. It is the first study to upgrade a specialized competency framework in a developing country and evaluate its construct and structural validity and reliability in the community setting. The sample size was sufficient for adequate statistical analysis for validation and assessment. The sample was also representative as it included pharmacists from all universities in Lebanon and pharmacists who graduated from outside Lebanon.

Nevertheless, a few limitations should be acknowledged. The snowball sampling technique could have led to selection bias. However, this risk is believed to be minimized since the sample included community pharmacists from all districts across Lebanon. Additionally, most participants were middle-aged pharmacists with an average of ten years of practice experience. Therefore, the competencies were not well-assessed among fresh graduates and older pharmacists. The length of the questionnaire may have been associated with exhaustion, ensuing in a possible risk of information bias, although it is believed that this risk is reduced as pharmacists were able to autosave their responses at any time and continue filling out the survey at their convenience. Finally, although a multivariable analysis was conducted to decrease confounding, residual confounding is still possible. Further studies are suggested to confirm the validity of the framework and expand its application to assessment situations.

## Conclusion

Our findings could demonstrate that the Lebanese specialized competency framework is a valid and reliable tool. The Lebanese community pharmacists reported being competent in all domains, with lower confidence in areas related to compounding, management, and emergency preparedness. Overall, higher competencies were positively associated with longer work experience and the number of received patients but not with the degree of education. This framework could be adopted by the authorities and implemented in the Lebanese community pharmacy setting. It could help assess the minimum competencies that community pharmacists should possess or acquire and direct initial and continuing education for better practice.

## Supplementary Information

**Additional file 1. **The specialized competency framework for community pharmacists' questionnaire.

## Data Availability

The datasets used and/or analyzed during the current study are available from the corresponding author on reasonable request.

## Abbreviations

FIP: International Pharmaceutical Federation
CPD: Continuing professional development
OPL: Order of Pharmacists of Lebanon
LIU: Lebanese International University
PCA: Principal component analysis
KMO: Kaiser–Meyer–Olkin
COVID-19: Coronavirus diseases 2019
