# Supplementary material for: The specialized competency framework for community pharmacists (SCF-CP) in Lebanon: validation and evaluation of the revised version
Source: J Pharm Policy Pract. 2023 Jun 21;16:77. doi: 10.1186/s40545-023-00585-6 (PMC10283199; doi:10.1186/s40545-023-00585-6)
Supplement: Supplementary file 1 — Additional file 1. The specialized competency framework for community pharmacists' questionnaire. [file 40545_2023_585_MOESM1_ESM.pdf]

نقابة صيادلة لبنان
Order of Pharmacists of Lebanon

INSPECT-LB
Institut National de Santé Publique, d'Épidémiologie Clinique et de Toxicologie
National Institute of Public Health, Clinical Epidemiology, and Toxicology

Advanced Competencies for Community Pharmacists

Dear pharmacist,

You are invited to participate in a survey about advanced competencies and skills acquired upon graduation of your highest degree related to your current field of work.

This study conducted by a group of academic researchers aims to determine the domains that need strengthening for an optimal-performing public health system.

Your participation in this study is voluntary and anonymous, and the information gathered in this 20-minute questionnaire will be treated confidentially. By completing it, you are consenting to participate in this study.

We thank you in advance for your time,

The research team.

Informed consent

Please check all the boxes to proceed to the survey

☐ I have read and understood the above information

☐ I understand that my participation is voluntary

☐ I understand that my data will be kept confidential

☐ I agree to participate in this study

DEMOGRAPHICS

1. **Age:**
2. **Gender:** ☐M ☐F
3. **Level of education:**
   ☐BS Pharmacy ☐PharmD/DPharm ☐Masters ☐PhD ☐Other:
4. **Highest degree related to your main field of work:**
   ☐BS Pharmacy ☐PharmD/DPharm ☐Masters ☐PhD ☐Other:
5. **Year of graduation from school/faculty of pharmacy:**
6. **University you graduated from as a pharmacist:**
   ☐UL ☐USJ ☐BAU ☐LAU ☐LIU ☐Other, country:
7. **University you earned your highest degree from:**
   ☐UL ☐USJ ☐BAU ☐LAU ☐AUB ☐LIU ☐Other, country:
8. **Language of pharmacy education:**
   ☐French ☐English ☐Other:
9. **Work Location:**
   ☐Beirut ☐Mount Lebanon ☐North Lebanon ☐South Lebanon ☐Beqaa
   ☐Currently not working
10. **Number of working days per week:**
11. **Number of working hours per day:**
12. **Number of patients received per day (community pharmacists only):**
    ☐<10 ☐10-50 ☐51-100 ☐>100
13. **Are you the owner of the pharmacy where you work?**
14. **How long (in years) have you been practicing as a community pharmacist?**
15. **Do you have another field of work? (Please select all that apply)**
    ☐ I do not have another field of work
    ☐Academia (teaching)
    ☐Preceptor
    ☐Clinical pharmacy
    ☐Research
    ☐Other:

COMMUNITY PHARMACIST COMPETENCIES

QUESTION: How confident are you in applying the below community pharmacist competencies?	Very confident	Fairly confident	Neither/ I don't know	Slightly confident	Not confident at all
**0 Fundamental Skills**	**0.1 Legal Considerations**				
**0.1.1** Apply laws and regulations that impact pharmacy practice.					
**0.1.2** Identify issues, pending legislation, and regulations across all levels of government.					
**0 Fundamental Skills**	**0.2 Product Procurement & Management**				
**0.2.1** Select and acquire products through the appropriate supply chain.					
**0.2.2** Anticipate, identify, and troubleshoot problems with the supply chain.					
**0.2.3** Manage inventory.					
**0.2.4** Handle drug waste.					
**0 Fundamental Skills**	**0.3 Compounding**				
**0.3.1** Compound extemporaneous preparations.					
**0.3.2** Perform elementary, non-sterile compounding					
**0 Fundamental Skills**	**0.4 Pharmacy Operation**				
**0.4.1** Managing the pharmacy operations efficiently.					
**0.4.2** Apply typical pharmacy dispensing workflow.					
**0.4.3** Comprehend and adopt a given set of pharmacy operating procedures.					
**0.4.4** Comprehend and adopt an existing collaborative drug therapy management system.					
**0.4.5** Evaluate prescription for legitimate medical use.					
**0.4.6** Prioritize and thoroughly complete tasks even with multiple interruptions occurring concurrently.					
**0.4.7** Implement dispensing processes when pharmacy automation is utilized.					
**0.4.8** Describe the roles and responsibilities of each pharmacy staff member[1].					
**0.4.9** Balance concrete specific motivation with constructive and professional criticism					
**0.4.10** Set and work towards meaningful goals with your staff					
**0.4.11** Allow training development by staff and supervisor					
**0.4.12** Work on the skills, attitudes, and values essential to the practice and profession					
**0.4.13** Monitor progress of the staff					
**0 Fundamental Skills**[2][3]	**0.5 Quality Improvement**				

**0.5.1** Optimize the concepts of quality measurement and improvement.					
**0.5.2** Apply national/international standards/guidelines/best practices related to your community pharmacy practice					
**0.5.3** Develop a plan for quality/performance improvement.					
**0 Fundamental Skills**	**0.6 Pharmacy Automation**				
**0.6.1** Operate a computerized system for dispensing medications					
**0.6.2** Understand the role of computerized pharmacy management systems in dispensing.					
**0.6.3** Dispense prescriptions utilizing technology-assisted workflow when applicable.					
**1 Safe and Rational Use of Medicines**	**1.1 Clinical Skills**				
**1.1.1** Demonstrate and routinely apply clinical skills and provide patient care services.					
**1.1.2** Ensure the optimal use of medicines.					
**1.1.3** Individualize therapy through the implementation of a patient's profile to the selection and modification of a medication regimen in collaboration with the prescriber					
**1.1.4** Describe and apply clinical practice guidelines to patient care.					
**1.1.5** Demonstrate knowledge of appropriate administration technique for dosage forms commonly dispensed in community pharmacy.					
**1.1.6** Describe common doses of drugs requiring monitoring and collaborative drug therapy management.					
**1.1.7** Proactively perform counseling and education which comply with current guidelines.					
**1.1.8** Assist patients with chronic diseases regarding appropriate use of chronic medications					
**1.1.9** Monitor for medications errors, drug interactions, and laboratory tests routinely					
**1 Safe and Rational Use of Medicines**	**1.2 Medication Therapy Management**				
**6.2.1** Develop a patient-centered, culturally responsive approach to medication management.					
**1.2.2** Define and appropriately document comprehensive MTM services.					
**1.2.3** Conduct a patient interview and provide education.					
**1.2.4** Conduct comprehensive medication review.					
**1.2.5** Identify and resolve medication therapy problems, manage drug interactions, and resolve gaps in care.					
**1.2.6** Recommend therapeutic alternatives and generic substitutions in collaboration with the prescriber					
**1.2.7** Document services and follow-up with other health professionals.					

**1.2.8** Use multiple MTM platforms as required by third-party payers and OPL.					
**1 Safe and Rational Use of Medicines**	**1.3 Compliance and Adherence**				
**1.3.1** Support and assist patient behavior change.					
**1.3.2** Identify and resolve patient-specific barriers to medication adherence.					
**1.3.3** Facilitate patient self-administration of medications and disease monitoring for minor ailments					
**1 Safe and Rational Use of Medicines**	**1.4 Problem-Solving/ Referrals**				
**1.4.1** Make appropriate recommendations or referrals.					
**1.4.2** Assess and resolve issues related to medication safety.					
**1 Safe and Rational Use of Medicines**	**1.5 Over-the-Counter Medicine**				
**1.5.1** Assist with patient self-care, including helping patients make appropriate selections of OTC medications.					
**1.5.2** Assist with patient self-care, including helping patients make appropriate selections of dietary supplements.					
**1.5.3** Assist with patient self-care, including helping patients make appropriate selections of herbal supplements.					
**1 Safe and Rational Use of Medicines**	**1.6 Pharmacovigilance**				
**1.6.1** Identify a potential adverse drug reaction					
**1.6.2** Consider that reporting an ADR is part of pharmacist duties					
**1.6.3** Demonstrate knowledge on reporting an adverse drug reaction to relevant authorities					
**2 Pharmacy Management**	**2.1 Functions**				
**2.1.1** Manage inventory costs and inventory levels or order points.					
**2.1.2** Identify cash flow problems and apply solutions to address.					
**2.1.3** Develop a business plan for clinical service programs.					
**2.1.4** Describe basic finance terms and analyze a financial statement.					
**2.1.5** Apply healthcare economics and pharmacoeconomics.					
**2.1.6** Describe strategies for asset protection and safety.					
**2.1.7** Use pharmacy technology effectively.					
**2 Pharmacy Management**	**2.2 Managed Care/Drug Coverage Policies**				
**2.2.1** Explain the general concept of managed care, associated with the benefit structure of a health plan.					
**2.2.2** Adapt best treatment strategies to patient socioeconomic status.					
**2.2.3** Provide guidance to patients seeking assistance to apply for drug payment programs.					
**2.2.4** Troubleshoot denied claims.					
**2.2.5** Discuss the concept of drug utilization review, formulary management and provide functional definitions of key managed					

care strategies (e.g., prior authorizations, step therapy, quantity limits).					
**2.2.6** Identify major factors that contribute to prescription drug-related fraud and abuse.					
**2.2.7** Identify major factors influencing drug costs for a managed care organization (e.g., pharmacy costs, drug pricing methodologies, contracts/rebates, discounts).					
**2.2.8** Meet payer requirements for reimbursement.					
**2.2.9** Reduce medication costs by providing less expensive alternatives if needed.					
**3 Professional Skills**	**3.1 Health Literacy**				
**3.1.1** Determine the patient level of health literacy by observation or interview.					
**3.1.2** Adjust counseling delivery and communicate at all levels of health literacy.					
**3.1.3** Solve adherence challenges created by low health literacy.					
**3 Professional Skills**	**3.2 Patient Communication**				
**3.2.1** Listen closely and attentively to patients.					
**3.2.2** Discuss pharmaceutical and other medical information thoroughly with patients and any family members.					
**3.2.3** Support patient behavior change through skills such as motivational interviewing.					
**3.2.4** Demonstrate respect for patient confidentiality and privacy rights.					
**3.2.5** Demonstrate patient compassion and empathy.					
**3 Professional Skills**	**3.3 Health Professional Communication**				
**3.3.1** Communicate effectively with colleagues, prescribers, and other healthcare providers					
**3.3.2** Contribute to effective interdisciplinary collaboration and integrated care.					
**3.3.3** Document appropriate therapeutic recommendations related to medication therapy.					
**3 Professional Skills**	**3.4 Team Communication**				
**3.4.1** Communicate with the pharmacy team, colleagues, prescribers and other care providers in an efficient manner.					
**3.4.2** Identify and manage conflict at all levels.					
**3.4.3** Supervise and motivate employees, staff, students, interns, residents.					
**3.4.4** Delegate tasks appropriately.					
**3.4.5** Articulate team objectives and measure and report team performance.					
**3 Professional Skills**	**3.5 Leadership Abilities & Personal Skills**				
**3.5.1** Display confidence in the patient care skills.					

**3.5.2** Demonstrate professional behavior (attitude, dress, appearance, etc.) in practice settings.					
**3.5.3** Embrace and advocate changes that improve patient care.					
**3.5.4** Be a gatekeeper to patient health.					
**3.5.5** Organize work and balance patient care and personal development.					
**3.5.6** Engage in regular professional development activities					
**3.5.7** Engage in professional organization activities					
**3 Professional Skills**	**3.6 Drug Information Skills**				
**3.6.1** Access and utilize appropriate drug information resources and provide an accurate and credible solution					
**3.6.2** Utilize a variety of drug-related reports, monographs, reviews, and policies using drug literature evaluation skills.					
**3.6.3** Evaluate the appropriateness of clinical trials and other study designs, including validation of methodology and assessment of data credibility.					
**3.6.4** Deliver timely drug information to the general public and other health professionals.					
**3.6.5** Implement career advancement through continuous professional development.					
**3 Professional Skills**	**3.7 Ethical Considerations**				
**3.7.1** Understand professional ethics as they apply to the practice of pharmacy.					
**3.7.2** Apply knowledge and understanding of ethical aspects of pharmacy practice required to evaluate a patient care decision.					
**4 Public health Fundamentals**	**4.1 Clinical Applications of Public Health**				
**4.1.1** Assess and support local and national health priorities and initiatives.					
**4.1.2** Educate population to access and understand health information on selection and rational use of medicines and other health products.					
**4.1.3** Participate in education and intervention in public health initiatives applicable to pharmacy practice.					
**4.1.4** Be knowledgeable about immunization schedules and requirements and actively involved in vaccination campaigns.					
**4.1.5** Collect, assess, and make recommendations based on the results of health and wellness screenings and diagnostic tests.					
**4.1.6** Promote healthy lifestyle and nutrition and describe how it impacts drug therapy and overall health/well-being.					
**4.1.7** Describe the role of a pharmacist in emergency situations.					
**4.1.8** Participate in the population-based provision of care (as distinguished from direct patient care).					
**5 Pharmacist Emergency Preparedness and Response (EPR)**	**5.1 Emergency Preparedness and Response**				
**5.1.1** Check for volunteering opportunities					

**5.1.2** Check for training opportunities					
**5.1.3** Address medication shortage and mitigation plan					
**5.1.4** Balance stockpile and availability of drugs for existing/chronic conditions					
**5.1.5** Partner with local authorities					
**5.1.6** Check for FDA/EMA Emergency Use Authorizations (EUAs) and expedited review and approval of tests/drugs for treatment					
**5.1.7** Follow actions and recommendations of local authorities					
**5.1.8** Involve trainees and staff in emergency response					
**5 Pharmacist Preparedness and Response in Emergency Situations**	**5.2 Operations Management**				
**5.2.1** Procure essential medications and supplies					
**5.2.2** Ensure medication delivery/safe storage					
**5.2.3** Develop workplace training and safety protocols (e.g., social distancing)					
**5.2.4** Secure PPEs or other needed materials					
**5.2.5** Monitor workers/assistants for symptoms					
**5.2.6** Adapt working hours to meet essential services during crises					
**5.2.7** Secure sanitizers and other medications when needed					
**5.2.8** Participate in interdisciplinary training to EPR teams					
**5 Pharmacist Preparedness and Response in Emergency Situations**	**5.3 Patient Care and Population Health Interventions**				
**5.3.1** Maintain patient confidentiality					
**5.3.2** Continue medication reviews, screening and/or testing/vaccination services safely					
**5.3.3** Identify at-risk populations					
**5.3.4** Educate patient about the ongoing crisis using evidence-based information and communications					
**5.3.5** Manage panic buying					
**5.3.6** Answer EPR-related calls					
**5 Pharmacist Preparedness and Response in Emergency Situations (EPR)**	**5.4 Evaluation, Research, and Dissemination for Impact and Outcomes**				
**5.4.1** Participate in research and studies on EPR					
**5.4.2** Publish and/or disseminate findings					
**5.4.3** Combat misinformation by disseminating evidence-based information to patients and sharing it on social media					
**5.4.4** Develop training programs for peers and other healthcare workers					

1. **What percentage of these competencies did you acquire during your undergraduate studies?**
2. **What percentage of these competencies did you acquire during your postgraduate studies?**
3. **What percentage of these competencies did you acquire from continuing education sessions?**
4. **What percentage of these competencies did you acquire by experience?**
